# Development of an Intervention to Create a Supportive Work Environment for Employees with Chronic Conditions: An Intervention Mapping Approach

**DOI:** 10.1007/s10926-020-09885-z

**Published:** 2020-03-21

**Authors:** A. R. Bosma, C. R. L. Boot, F. G. Schaafsma, G. Kok, J. R. Anema

**Affiliations:** 1 Department of Public and Occupational Health, Amsterdam Public Health Research Institute, Amsterdam UMC, VU University Amsterdam, Amsterdam, The Netherlands; 2 Faculty of Psychology and Neuroscience, Maastricht University, Maastricht, The Netherlands

**Keywords:** Work, Chronic disease, Occupational health services, Organizations

## Abstract

*Purpose* This study describes the development of an evidence-based intervention to create a supportive work environment for employees with chronic conditions. Occupational physicians (OPs) play an important role in guiding organizations in this process of organizational change. Supportive work environments can aid in preventing work-related problems and facilitate sustainable employment. Current workplace interventions for employees with chronic conditions are mainly focused on return to work or a reduction in sick leave at the individual worker’s level. This study contributes to the literature an organizational-level intervention which utilizes a preventive approach. *Methods* Intervention mapping (IM) is a six-step, structured protocol that was used to develop this intervention. In step 1, a needs assessment was conducted to define the problem and explore the perspectives of all stakeholders involved. The program outcomes and the performance objectives of employees with chronic conditions and occupational physicians were specified in step 2. In step 3, appropriate methods and practical applications were chosen. Step 4 describes the actual development of the intervention, consisting of (1) a training for occupational physicians to teach them how to guide organizations in creating a supportive work environment; (2) a practical assignment; and (3) a follow-up meeting. The intervention will be implemented in a pilot study in which occupational physicians will put their acquired knowledge and skills into practice within one of their organizations, which is delineated in step 5. *Conclusions* IM proved to be a valuable and practical tool for the development of this intervention, aiming to facilitate sustainable employment for employees with chronic conditions.

**Electronic supplementary material:**

The online version of this article (10.1007/s10926-020-09885-z) contains supplementary material, which is available to authorized users.

## Introduction

The number of people in the working population with one or more chronic conditions is increasing [1, 2]. Work participation rates among those with a chronic condition are lower compared to participation rates of the general population. Working with a chronic condition can lead to certain physical or psychological challenges, possibly resulting in sick leave or job loss. Prevention of work-related problems, sick leave, and job loss among these employees is of great importance since returning to work has proven to be difficult [2, 3].

Much research has been conducted on factors associated with sustainable work participation for employees with chronic conditions, showing that personal, disease-related, as well as work-related factors are of importance [4–6]. In the last decade, a wide variety of interventions have been developed to support people with chronic conditions in their work in order to prevent productivity loss, sick leave, or job loss. However, these interventions, addressing factors such as work accommodations, empowerment, and self-management, have shown only limited effects [7–10].

In recent years, people with chronic conditions have been encouraged by the Dutch government and society to take control over their lives, including their work [11]. Self-control is a concept that relates to controlling one’s responses and behaviors with the purpose of reaching long-term goals [12, 13]. An interplay between impulse control, deliberate decision making, and the availability of certain cognitive resources underlie the behavior that is carried out. One’s level of self-control can be seen as a benchmark for adaptation [12, 14]. Although self-control is often described in relation to health behaviors (e.g. healthy eating) [15], it may also aid workers with adjusting to the new circumstances of working with a chronic condition. Using Huber’s new definition of health, “having the ability to adapt and self-manage” (p. 2) [16], having higher levels of self-control at work and the possibility of exerting self-control might improve wellbeing and health, thereby facilitating sustainable employment for employees with chronic conditions.

Interventions aimed at increasing the exertion of self-control can focus on an individual’s *capacity* to exert self-control or on changing the *context* in which self-control is exerted [17]. Based on available literature, it is clear that a person’s level of self-control can be increased through training and practice [14]. However, a meta-analysis of the effect of self-control training shows only a minor effect [18]. Changing the context in which self-control can be exerted has been shown to be a more successful strategy in changing the desired behavior [19]. This implies that employees with chronic conditions are more likely to exert self-control in a supportive work environment where they feel enabled to do so.

Occupational health professionals could play a key role in increasing the exertion of self-control of employees with chronic conditions, both by supporting individual employees and by helping to create these supportive work environments. In the Netherlands, occupational physicians (OPs) have the task of supporting and advising employees and organizations on issues related to work and health to facilitate sustainable employment [20]. In recent years, the Dutch government has emphasised the role of OPs in the prevention of work-related problems, by obligating organizations to ensure their employees access to preventive consultation hours with OPs [21]. The preventive role of OPs remains small, however, and they mainly deal with employees with existing problems and cases of absenteeism [22, 23]. This is unfortunate given that OPs also have the desire to focus more on prevention [24]. Dutch occupational health and safety legislation stipulates that in case of work-related problems or sick leave, both the employer and employee must take responsibility for securing healthy and sustainable employment [25, 26]. As a consequence of this shared responsibility, the distance of OPs to the organization is increasing, making them less visible as advisors to employers on health and the prevention of work-related problems within organizations.

Based on the importance of the context in which self-control is exerted, it can be inferred that it is essential that an intervention for employees with chronic conditions should focus on changing the work environment. These interventions aimed at organizational change can result in creating supportive environments, thereby providing employees with chronic conditions with the right conditions to exert self-control and leading to the prevention or early identification of work-related problems. By changing the OP’s role and making OPs an essential part of organizational-change interventions, they are able to use their expertise on work and health to guide organizations in creating supportive work environments for employees with chronic conditions. This role enables OPs to collaborate closely with organizations, reducing the distance between employer and OP and supporting and guiding preventive measures within an organization. To the best of our knowledge, no intervention has been developed aimed at increasing the exertion of self-control for employees with chronic conditions by changing the work context. The aim of this study is to develop an intervention for OPs with the purpose of creating supportive work environments for employees with chronic conditions by guiding organizations in making these changes. For the development process, we used intervention mapping (IM), which “provides a framework for effective decision making during planning of intervention programs, including the planning of implementation and evaluation” [27].

## Intervention Mapping Process

**Fig. 1:**
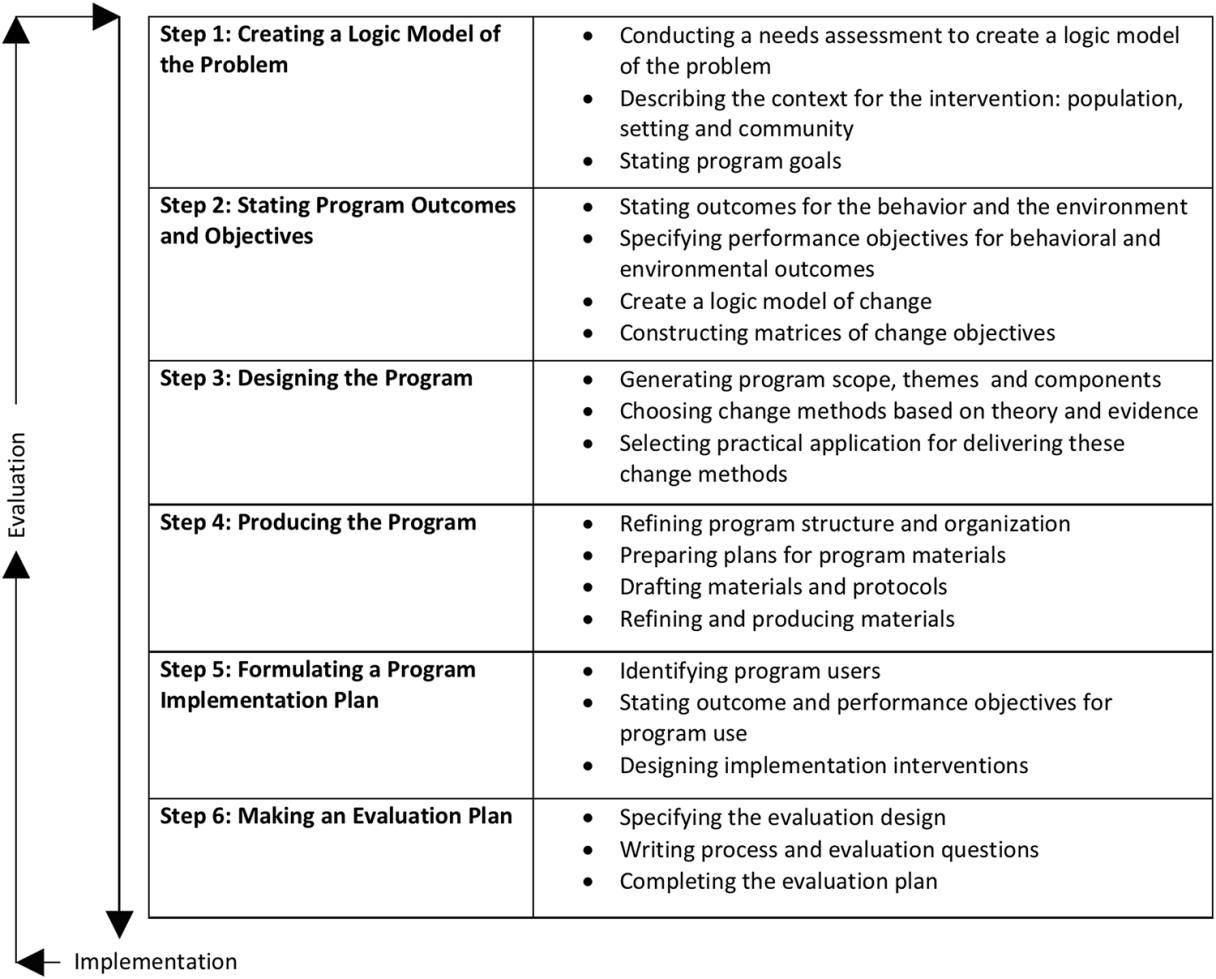
The six-step intervention mapping protocol. Adapted from Ref. [27]

IM is a stepwise protocol used for planning and developing effective behavioral and environmental change interventions, consisting of six steps (presented in Fig. 1). The iterative nature of the IM protocol stimulates the use of theory as well as existing and newly-acquired evidence for the intervention development, with the flexibility to go back and forth through the different steps. Involving stakeholders in the process enables the interventions to be fit to the needs and wishes of all involved [27]. The relevant stakeholders in this study are OPs, employees with chronic conditions, and organizational representatives (e.g. supervisors/management, co-workers, and the human resources department within the organization). The project team involved in the development of this intervention consisted of two health scientists and two OPs. An IM expert advised the project team during the development process.

### Step 1: Logic Model of the Problem

In the first step of the IM process, a logic model of the problem was created which helped in defining the problem and depicting a representation of the causal relationship between the problem and its causes. As part of this step, a needs assessment was conducted with the aim of assessing the nature and extent of the problem (‘what is’) and the needs (‘what should be’) of all the stakeholders. The needs assessment consisted of a qualitative synthesis to gain insight into the concept of self-control and the influence of the environment on the exertion of self-control for employees with chronic conditions [28]. In addition, the literature was reviewed and interviews were held with all relevant stakeholders (employees with chronic conditions, OPs, as well as organizational representatives including employers and human resource managers) to capture a complete overview of perspectives.

#### Employees with Chronic Conditions

Self-control is defined as “the capacity for altering one’s own responses, especially to bring them into line with standards such as ideals, values, morals, and social expectations, and to support the pursuit of long-term goals” (p. 351) [13]. In our study, sustainable employment is considered the long-term goal for employees with chronic conditions. Limited research has been conducted on self-control in the context of work, focusing instead on organizational management [29, 30]. The literature available on self-control was not able to provide us with an understanding of which specific behaviors employees with chronic conditions should alter or express to help them reach the long-term goal of sustainable employment. Therefore, we explored available international qualitative literature which examined factors enabling sustainable employment, specifying the desired self-control behaviors important for preventing work-related problems and the influence of the environment on the enactment of these behaviors. Four self-control behaviors from the perspective of employees with chronic conditions emerged from this qualitative synthesis: (1) disclosure, (2) finding a healthy balance, (3) requesting work accommodations and support, and (4) management of symptoms and limitations in the workplace. Disclosure of the condition at work can create understanding and support among co-workers, supervisors, and employers, and can facilitate both better management of the condition and the implementation of accommodations. Disclosure is influenced by the personal beliefs of the employee as to possible consequences of disclosure, disease-related factors such as predictability and invisibility of the disease, and workplace factors, including workplace culture and the employee’s relationship with their co-workers, supervisors, and employers. Finding a healthy balance is important for enabling employees with chronic conditions to continue working and relates to the decision-making process of an employee with a chronic condition. Employees make decisions in relation to strategies that make sustainable work participation possible, such as energy management or a job change. Requesting accommodations and support is crucial for fitting the job to the capacities of the employee and adjusting to the chronic condition and limitations at work. An accepting and supportive work environment makes it easier to ask for support and accommodations. Management of symptoms and limitations in the workplace enables sustainable work productivity. This requires an awareness of symptoms, an acceptance of the chronic condition and its limitations, and for the employee to take responsibility for managing these symptoms and limitations. Based on this synthesis, the work environment appears to have an important influence on employees expressing these self-control behaviors. An accepting workplace culture and an understanding and trusting relationship with co-workers, supervisors, and employers facilitates the exertion of self-control (e.g. by lowering the threshold to disclose the condition). The main findings of the interviews with employees with chronic conditions underscored the same self-control behaviors found in the qualitative synthesis and emphasized the importance of a supportive work environment in enabling the expression of these behaviors. In addition, employees spoke of a lack of knowledge, lack of organizational policies, and lack of compliance to organizational regulations, all of which made disclosure and acquiring work accommodation more difficult.

#### Occupational Physicians

During the interviews, OPs expressed the importance of early identification of work-related problems. Currently, OPs do not have a clear overview of all employees with chronic conditions within the organization, as most employees with chronic conditions consult their OP only in cases of already existing work-related problems or sick leave. Support from and collaboration with the work environment were described by OPs as important prerequisites for sustainable employment for employees with chronic conditions. OPs also stated the importance of a positive organizational attitude towards employees with chronic conditions and a supportive workplace culture. In a study by Abma et al., OPs also stated the importance of clear communication and a supportive organizational culture on sustainable employment. In addition, OPs described their desire to have a more preventive role, instead of focusing a large proportion of their time on return to work or sick leave [24].

#### Organizational Representatives

During the interviews, employers and human resource managers highlighted the importance of collaborating with OPs and employees and making sure that mutual expectations are clear. Currently, employers and human resource managers mainly focus on looking at an individual employee’s work capacity and, in case of need, facilitating work adjustments. Work adjustments can, however, only be implemented to a certain extent. Adjustments such as task redistribution or shifting work tasks to colleagues is not always possible. OPs and organizational representatives pointed out the importance of having a clear organizational policy for working with a chronic condition and preventing work-related problems within an organization. Literature on the needs and perspectives of organizational representatives has shown the important role of employers and human resource managers when it comes to supporting employees with chronic conditions [31, 32]. At the same time, the literature describes a lack of knowledge and awareness among human resource managers and line-managers of the impact of a chronic condition on working life [33, 34]. Having a clear company policy, providing early support and accommodations, facilitating good cooperation between managers and employees, and having employees take responsibility (e.g. communicating to managers and making decisions) are some of the factors indicated by employers and human resource managers as facilitating sustainable employment [24, 35].

The results of this first step provide clarity on the behaviors of an employee with a chronic condition, the influence of the environment (work environment and current support from OPs) and perspectives of OPs and organizational representatives on sustainable employment. It is clear that preventing work-related problems and sustainable employment requires the commitment of all stakeholders involved. Employees exerting self-control means executing the abovementioned desired behaviors. However, employees must be enabled and supported by the work environment to actually execute these behaviors. Organizational policies could thereby facilitate sustainable employment for employees with chronic conditions. OPs can fulfil their preventive tasks by offering advice on organizational policy development and guiding organizations towards more supportive work environments.

### Step 2: Program Outcomes and Objectives—Logic Model of Change

In this second step, a logic model of change was created (see online resource 1), visualizing the effects of the intervention on behavior and the environment. As a starting point, the behavioral outcome (*the employee with a chronic condition will exert self-control*) of the intervention to be developed was identified, after which the performance objectives were specified. The performance objectives operationalize what needs to be done in the behavior of the employee with a chronic condition in order to accomplish the behavioral outcome. The performance objectives associated with the behavioral outcome of the employees with chronic conditions are listed in Table 1. These performance objectives are based on the four self-control behaviors described in the needs assessment.

**Table 1:** Performance objectives for an employee with a chronic condition

1. Decide in which cases disclosure of the chronic condition could be of help for sustainable employability and follow-up on these decisions (designating when to disclose, to whom and what information)
2. Ask for adjustments and support from employers, co-workers, the social environment, and health care providers
3. Make decisions with the aim of fitting the job to personal capacities and to maintain balance in life and follow-up on these decisions
4. Manage limitations and respond to symptoms at work

Environmental outcomes can be categorized into different levels, including interpersonal, organizational and community levels. In this organizational intervention, the focus was on the environmental outcome at the organizational level (*OPs collaborate with the work environment in supporting employees with chronic conditions to exert self-control*), which was considered the most relevant environmental outcome level. Performance objectives were identified for the environmental outcome, with OPs being the environmental agents of importance at the organizational level (see Table 2). These performance objectives show the direct collaboration between OPs and an organization for creating an organizational policy and supportive work environment, thereby indirectly supporting employees with chronic conditions to exert self-control.

**Table 2:** Performance objectives for OPs (environmental agents)

1. OP creates awareness within the organization of the need for an organizational policy to facilitate employees with chronic conditions staying at work
2. OP guides the employer in exploring organizational barriers which inhibit employees with chronic conditions from exerting self-control
3. OP guides the employer in exploring possible solutions for these organizational barriers which inhibit employees with chronic conditions from exerting self-control
4. OP helps to create an organizational policy and a supportive work environment to facilitate the ability of employees with chronic conditions to exert self-control and stay at work

Determinants are factors underlying the performance of behavior. The needs assessment provided information on personal determinants on the behavioral and organizational levels that are associated with the performance objectives of employees with chronic conditions and the performance objectives of OPs, respectively. Based on the determinants yielded from the needs assessment and the determinants described in behavior change theories (e.g. Reasoned Action Approach [36]), attitude, skills and self-efficacy, and perceived norms were selected. Subsequently, matrices of change objectives were constructed for the behavioral outcome as well as for the environmental outcome by combining performance objectives with determinants for employees and OPs. Change objectives operationalize what employees with chronic conditions as well as OPs participating in the program need to learn or change to meet and/or maintain the performance objectives. Examples of matrices of change objectives for the behavioral outcome and environmental outcome are shown in online resource 2.

### Step 3: Program Design

The intervention was conceptualised and designed in step 3, based on the logic model of change created in step 2. In this step, an initial program plan was conceived with the program components, scope, and sequence. Additionally, theory- and evidence-based methods and practical applications were chosen to influence the change objectives.

The design of the intervention and selection of chosen methods and applications were extensively discussed within the project team to make sure that appropriate methods were used to influence the relevant determinants. As the work environment is crucial for employees with chronic conditions to express the desired self-control behaviors in the workplace (e.g. disclosure and requesting accommodations), the scope of the program was to develop an organization-specific policy and to create a supportive environment for these employees. By changing the role of OPs, they are able to focus more on prevention of work-related problems and support of organizational preventive actions. OPs can fulfil their preventive tasks by guiding and advising organizations in the process of organizational policy development and creating a supportive work environment. Online resource 3 shows examples of the theoretical methods and practical applications chosen for changing the attitudes, skills and self-efficacy, and perceived norms among the OPs, enabling them to guide organizations in developing an organizational policy and creating a supportive work environment.

When developing an organizational policy and creating a supportive work environment, it is important to include all stakeholders within an organization in the process. The participatory approach (PA) is an effective evidence-based approach for addressing and tackling existing barriers in an environment where different stakeholders could have varying perspectives regarding these barriers. The PA is a structured six-step process: (1) creating the right conditions, (2) analysis of barriers, (3) analysis of solutions, (4) plan of action, (5) implementation, and (6) evaluation. It can be used at both the individual or the organizational level to facilitate sustainable employment and the health of employees in an organization [37–39]. In this study, the PA will be used by OPs and applied at the organizational level to develop an organizational policy for employees with chronic conditions and to create a supportive work environment. When applying the PA at the organizational level, OPs, employees with chronic conditions, and all other relevant organizational representatives (e.g. supervisors, human resource managers) should be involved in the process. The likelihood of successful organizational change is improved by the joint effort of all relevant stakeholders within the organization.

Having a process leader to guide all the stakeholders through the different steps is essential when applying the PA. As OPs are considered suitable professionals to guide an organization into a supportive work environment for employees with chronic conditions, the plan was to train OPs in serving as a process leader when applying the PA in an organization. In addition to the knowledge and skills of the PA, it is also essential for the OPs to understand the concept of self-control and the associated self-control behaviors. An understanding of the influence of the work environment on the expression of self-control behaviors by employees with chronic conditions is also essential. OPs can use this knowledge to create awareness within the organization and to provide organizational representatives with information during the PA process.

### Step 4: Program Production

The methods and practical applications chosen in step 3 were operationalized into the final program in step 4. The structure and organization of the program were explained in a protocol, program materials were developed, and existing materials were reviewed and adapted as needed to address the change objectives.

The program we developed consists of a training, a practical assignment, and a follow-up meeting for OPs. It is suitable for all OPs, whether they are self-employed, working for an occupational health services agency, or working within the occupational health services department of an organization. The training provides the OPs with (a) theory and evidence on the self-control behaviors of employees with chronic conditions and the importance of a supportive work environment in expressing these self-control behaviors, and (b) information on how to apply the PA and act as a process leader in an organization in order to help the organization create organizational policy and a supportive work environment. During the training, theory on self-control behaviors and the PA will be alternated with short exercises, giving the OPs the opportunity to practice certain steps of the PA. Additionally, these exercises offer ways to reflect on the level of exertion of self-control behaviors in the organization the OP is working in. The training will be given by two members of the project team. At the start of the training, the participating OPs will receive a training manual containing (1) practical information, (2) the slides of the PowerPoint presentation to be used during the training, (3) information on the practical assignment, and 4) background information. At the end of the training, the OPs will receive further instruction on the practical assignment.

In the practical assignment, the OPs will need to apply the six steps of the PA in one of the organizations they are working for. OPs will start with creating the right conditions for applying the PA in the organization, one of which is creating a working group with employee and organizational representatives. The OP will serve as a process leader to guide this working group during three meetings. During the first meeting, the workgroup members will analyze and identify existing barriers inhibiting the execution of self-control behaviors within their organization. The second meeting will be used for brainstorming solutions for the identified barriers and a plan of action for the implementation of these solutions. OPs will thereafter monitor the implementation of these solutions within the organization. These solutions provide input for organizational policy and contribute to the creation of a supportive work environment. During the third meeting, the implemented solutions will be evaluated. Forms have been developed for guiding the PA process during the practical assignment. These forms are included in the manual (see online resource 4 for examples). Six months after the training, a follow-up meeting will be planned in which experiences with the practical assignment will be shared between the OPs.

### Step 5: Implementation Plan

Considering program implementation began in step 1 and extended to step 5. In step 5, a plan for the implementation of the program was developed specifying the potential implementers of the program. Program outcomes and performance objectives for adoption, implementation, and maintenance were written, after which matrices of change objectives for implementation were constructed. After selecting the proper change methods and applications, a strategy for adoption, implementation, and maintenance was designed.

Implementation of the program will occur in a pilot study in which the practical assignment will be used to explore the usability, practicality, and effectiveness of the program. OPs who participated in the training will put their knowledge and skills from the PA into practice in one of their organizations. Two important program outcomes were identified prior to the start of the pilot study: (1) the organization is positive about developing an organizational policy and creating a supportive work environment with use of the PA and (2) OPs are able to carry out the PA for the development of this organizational policy. Since this program aims to include all relevant stakeholders in the process, OPs as well as the organizations (including employees and relevant organizational representatives) are important to the successful implementation of the program. However, OPs and employers are considered the most relevant implementers because of their responsibility for initial implementation actions. Therefore, performance objectives for both these environmental agents (OPs and employers) are specified (see Tables 3 and 4).

**Table 3:** Performance objectives for implementation by OPs (environmental agents)

1. OP identifies relevant stakeholders within the organization (e.g. employees with chronic conditions, supervisors, human resources managers)
2. OP makes the sense of urgency of implementing organizational policy clear to the relevant stakeholders
3. OP explains and convinces the employer of the added value of the PA for the development of organizational policy
4. OP initiates the start of the PA in the organization
5. OP guides the organization through the PA process

**Table 4:** Performance objectives for implementation by the employer (environmental agents)

1. The employer supports the development of an organizational policy for employees with chronic conditions
2. The employer approves the use of the PA for the development of an organizational policy
3. The employer facilitates the PA by providing man hours and financial means
4. The employer actively participates in the PA for the development of an organizational policy

For this pilot study, OPs were targeted through the Netherlands Society of Occupational Medicine and a large Dutch occupational health services agency, and were invited to participate in the program. All OPs working for an organization which they thought might be open to implementing the program were eligible for participation. Since OPs were targeted instead of organizations, it was unclear in advance what type of organizations would ultimately participate in the program. OPs working for a variety of organizations were willing to participate, including organizations in the health care, financial, logistics, industrial and cultural sectors. Since the program developed for this pilot is a universal intervention, it can be implemented in any organization regardless of size, work sector, or the current number of employees with chronic conditions. Given the large portion of the population living with one or more chronic conditions, it was expected that the majority of organizations would have at least some employees with chronic conditions. Prior to the training, participating OPs were sent preparatory questions, the answers of which could be used to further tailor the training to the needs of the participants.

Given that each organization has a different structure, relevant organizational representatives to involve in the program can differ. Identification of relevant stakeholders within the organization by OPs is therefore a first step in the implementation phase. In order for employers to support the development of an organizational policy and organizational change they need to be aware of the importance of such a policy and the influence that a supportive work environment can have on employees with chronic conditions. At the start of the implementation phase, OPs were advised that members of the project team could assist in highlighting the urgency of an organizational policy and supportive work environment and explaining the added value of the PA to the organization (performance objectives 2 and 3 for the OPs).

### Step 6 Evaluation Plan

In the final step of the IM protocol, a plan for evaluating the effectiveness of the program on the change objectives and the actual behavior was developed. Results of this evaluation are expected in 2021.

## Discussion

This study describes the systematic development of a program for OPs using the IM protocol. The program consists of a training, a practical assignment, and a follow-up meeting for OPs. The program aim is to develop an organizational policy and create a supportive work environment for employees with chronic conditions thereby enabling them to exert self-control.

Targeting the workplace has been a focus of many interventions aimed at maintaining health and employment among employees, either on the individual employee level or on the organisational level. Workplace interventions have been developed focusing on issues such as improving employees’ lifestyles (e.g. sitting time or nutrition) or preventing work-related stress and injuries [40–43]. In the last decade, numerous workplace interventions have also been developed to prevent work disability for employees with chronic conditions [44–47]. When taking a closer look at these workplace interventions, three things stand out. First, the majority of these interventions have focused on employees on sick leave and strategies for reduction in the duration of absences and for returning to work [45, 46, 48, 49]. The number of interventions aimed at actually preventing work-related problems and promoting sustainable employment for employees with chronic conditions is lacking [50]. Second, a large proportion of interventions are directed at employees with psychological or musculoskeletal disorders [51]. Finally, prevention-focused interventions aimed at sustainable employment for employees with chronic conditions are almost always directed towards the individual employee instead of the organization as a whole, including stakeholders within the work environment [52, 53]. Different aspects of an organization can be targeted in organizational-level interventions, such as job demands, work conditions, or psychological or social factors (e.g. organizational support). Changing organizational culture and support is challenging but interventions based at the organizational-level have been shown to provide a more sustained effect on employees’ health in comparison to individual-level interventions [47]. The intervention described in this study adds to the literature an innovative, organizational-level intervention with a preventive approach which is aimed at employees with different types of chronic conditions.

With the growing number of employees with chronic conditions, a greater focus on prevention and sustainable employment within organizations is essential. Organizations differ in their ways of dealing with employees with chronic conditions with regard to the level of support offered, including the realization of work accommodations [54]. A negative attitude towards employees with chronic conditions, not knowing how to support and accommodate these employees, and lack of organizational policy related to things like work accommodation can all contribute to this problem. In addition, a country’s occupational health and safety legislation influences the way employers respond to these employees [34, 54, 55]. This same legislation also delineates the roles and responsibilities of occupational health professionals and their subsequent tasks [56, 57]. Despite the renewed Dutch labor legislation and focal point of prevention in the mission statement of the Netherlands Society of Occupational Medicine, prevention in occupational health care remains difficult to enact [58]. With their pivotal role in occupational health care, OPs have the expertise and ability to encourage and support employers with preventive actions and strategies for work-related problems that employees with chronic conditions may have. By positioning OPs as process leaders during the PA in this intervention, they are in a better position to play a preventive role.

The use of OPs as process leaders in this intervention also has limitations. Firstly, the intervention was tailored to the role of OPs in the Dutch context. In various other countries, the role of OPs differs from the role of Dutch OPs [23, 59]. In these other countries, however, different occupational health professionals such as occupational health nurses, return to work coordinators, or organizational psychologists could also fulfill the tasks of process leader [60, 61]. Secondly, occupational health care by OPs in the Netherlands is not freely accessible to all types of workers [62]. Self-employed workers, making up 12% of the Dutch working population, are not able to use the services offered by OPs [63], making the intervention not applicable to this group of workers. On the other hand, since this intervention is aimed at changes at the organizational level, all workers within an organization are able to benefit from the changes. This includes temporary agency workers within an organization, who, according to Dutch laws, otherwise would not have access to OPs.

### Methodological Considerations

Workplace interventions are complex, with numerous stakeholders involved. IM proved to be a valuable tool for the systematic development of this intervention, with several underlying reasons for the practicality of this approach. IM provided us with a structure to start sorting out the causal relationships of the problem and finding out the needs of all stakeholders involved. Based on the causal relations and stakeholders’ needs identified in the IM steps, it was clear what changes were necessary. Evidence-based decisions could thereby be made to focus the intervention to match the context in which it must be implemented. Since the program was initiated to support the development of knowledge and skills of OPs, our initial thought was to develop an intervention focusing on the OP. However, the evidence gathered in the IM steps shifted the focus of the intervention and its implementation to the work environment in which OPs would need skills as process leaders. Additionally, IM also provided an understanding of the complexity of the context, guidance on deciding what methods to use, and subsequent practical applications. However, although IM was used to optimize the development of the intervention, some drawbacks of this method could be identified. Following all the steps of the protocol is a time-consuming process. Furthermore, although IM aids in optimizing the effect of the intervention, using IM is not a guarantee for success, as pointed out by the review of Fassier et al. [64]. In this study, the needs assessment (as a first step in the IM protocol) showed the causal relations of the problem. In addition to the employees and other actors within the work environment, the health care environment and the social environment both influenced the possibility of employees with chronic conditions exerting self-control. Because the work environment was of primary importance, the health care and social environments were not targeted in this intervention. Adding elements to the program aimed at influencing the health care and social environments could further improve the effects of the program. IM contributed to the development of a clear implementation and evaluation plan.

### Practical Implications

Changing employees’ behavior is difficult, especially when optimal conditions for carrying out certain behaviors are absent. The same applies for self-control of employees with chronic conditions. Creating a supportive and understanding work environment provides these employees with the ability to exert self-control, while an organizational policy will provide the organization with clear procedures for employers and employees on addressing the prevention of work-related problems. The intervention developed in this study provides OPs with the necessary skills to serve as process leaders in the development of organizational policy and creating supportive and understanding work environments. An optimal work environment for the expression of self-control behaviors can lead to early identification or prevention of work-related problems among employees with chronic conditions and sustainable employment. This will benefit both employees with chronic conditions as well as employers. Once proven effective after the pilot study (expected results in 2021), this program could be imbedded in educational programs for OPs.

### Research Recommendations

It is to be expected that the effectiveness of the intervention will vary for different work settings. Aspects such as the size of the organization, the number of management layers and types of employees (e.g. white or blue collar) could influence the effectiveness of the intervention. Further research should be conducted to investigate contextual factors and the optimal conditions for implementing interventions in the workplace. The possibility of targeting organizations instead of OPs could also be explored.

## Electronic supplementary material

Below is the link to the electronic supplementary material.

Supplementary file1 (PDF 213 kb)

Supplementary file2 (PDF 274 kb)

Supplementary file3 (PDF 182 kb)

Supplementary file4 (PDF 349 kb)

## Abbreviations

CO: Change objective
IM: Intervention mapping
OP: Occupational physician
PA: Participatory approach
