# Supplementary figures and images for: Development of an Intervention to Create a Supportive Work Environment for Employees with Chronic Conditions: An Intervention Mapping Approach
Source: J Occup Rehabil. 2020 Mar 21;30(4):624–34. doi: 10.1007/s10926-020-09885-z (PMC7716852; doi:10.1007/s10926-020-09885-z)

## Online resource 1 Logic Model of Change

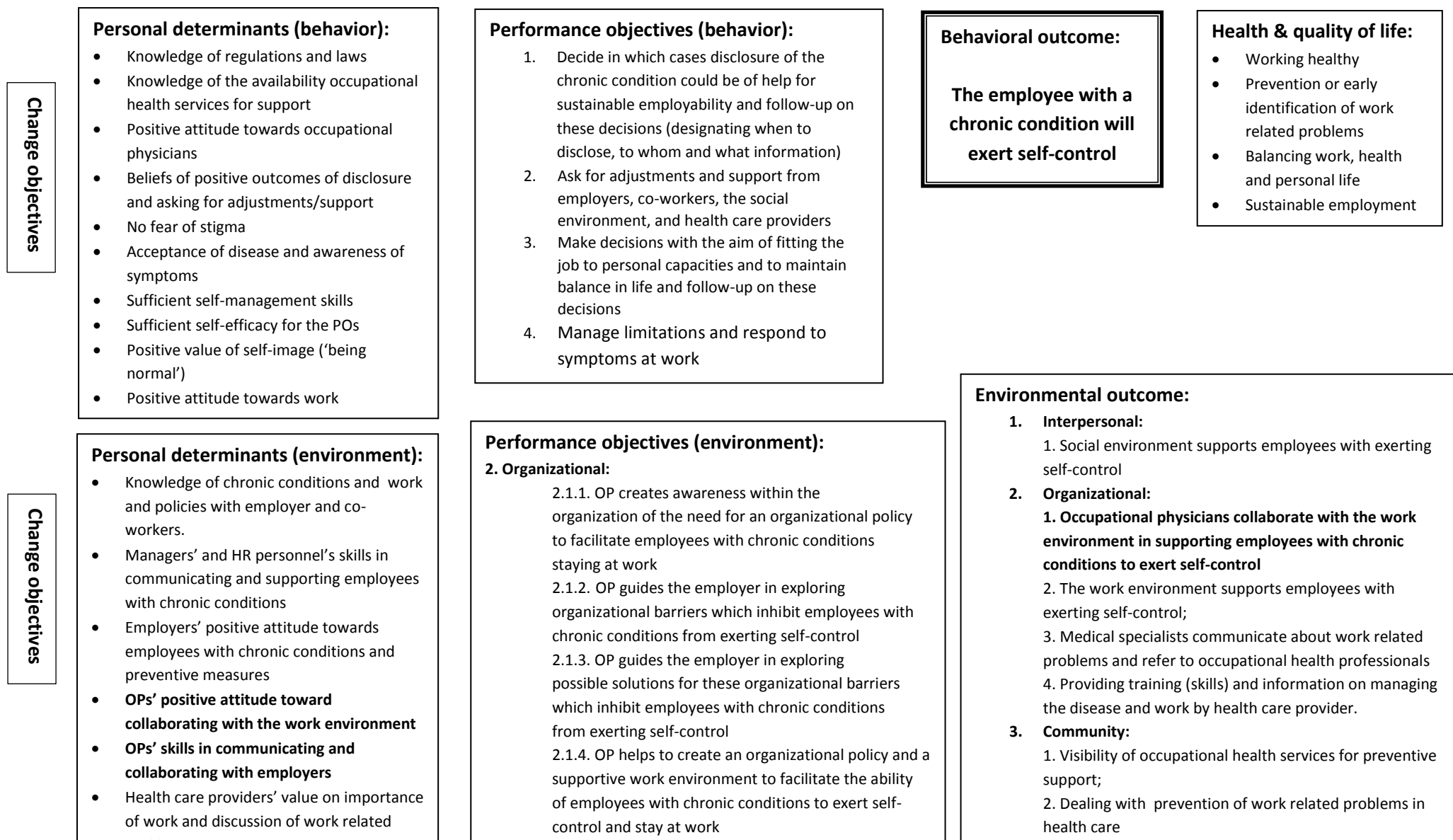

Supplement: Supplementary file 1 — Supplementary file1 (PDF 213 kb) [file 10926_2020_9885_MOESM1_ESM.pdf]
