# Supplementary material for: Development of an Intervention to Create a Supportive Work Environment for Employees with Chronic Conditions: An Intervention Mapping Approach
Source: J Occup Rehabil. 2020 Mar 21;30(4):624–34. doi: 10.1007/s10926-020-09885-z (PMC7716852; doi:10.1007/s10926-020-09885-z)
Supplement: Supplementary file 2 — Supplementary file2 (PDF 274 kb) [file 10926_2020_9885_MOESM2_ESM.pdf]

Online resource 2

Table 1 An example of a matrix of change objectives for employees with chronic conditions

**Behavioral outcome: The employees with a chronic disease will exert self-control**			
**Performance objectives**	**Personal determinants**		
	**Attitude**	**Skills & Self-efficacy**	**Perceived norm**
1. Decide in which cases disclosure of the chronic condition could be of help for sustainable employability and follow-up on these decisions (designating when to disclose, to whom and what information)	A1a. Express positive feelings about disclosing the condition and sharing information about the impact of working with the condition to work environment<br>A1b. State that disclosure helps in gaining adjustments and support	S1a. Express confidence in the ability to talk about the condition at work<br>S1b. List strategies that can help with talking about the condition at work<br>S1c. Describe what information will be shared and to whom	P1a. Perceive the employer to be positive about disclosing the condition<br>P1b. Feel support from employer and colleagues to disclose the condition
2. Ask for adjustments and support from employers, co-workers, the social environment, and health care providers	A2a. Express positive feelings about receiving support and adjustments in maintaining employment<br>A2b. State that asking for adjustments and support helps in fitting the job to the capacities and adjust to the condition at work	S2a. Express the confidence of recognizing the need for adjustments and support<br>S2b. Lists the available people (employers, co-workers, social environment, occupational physician and health care providers) and options for support<br>S2c. Lists the laws and policies that protect and facilitate employees with chronic conditions	P2a. Recognize that the employer is positive about asking for adjustments and support<br>P2b. Feel support from employer and colleagues to ask for adjustments and support<br>P2c. Feel support from policy and legislative system to ask for adjustments and support
3. Make decisions with the aim of fitting the job to personal capacities and to maintain balance in life and follow-up on these decisions	A3a. Express positive feelings about making decisions<br>A3b. State that decision making can help with energy management and	S3a. Express confidence about the ability to weigh the options and make the right decisions	P3a. Recognize that the employer is positive about making decisions that help to find the right balance in life<br>P3b. Feel support from employer and colleagues to

 A.R. Bosma[1], C.R.L. Boot[1], F.G. Schaafsma[1], G. Kok[2], J.R. Anema[1]; [1] Department of Public and Occupational Health, Amsterdam Public Health Research Institute, Amsterdam UMC, VU University Amsterdam, Amsterdam, The Netherlands. [2] Faculty of Psychology and Neuroscience, Maastricht University, Maastricht, the Netherlands; a.bosma@amsterdamumc.nl

	maintaining balance in work, health and personal life	S3b. Lists his/her priorities with regard to work, health and personal life<br>S3c. List the needed information for decision making	make decisions that help to find the right balance in life
4. Manage limitations and respond to symptoms at work	A4a. Express positive feelings about taking responsibility to manage the symptoms and limitations at work<br>A4b. State the consequences of not managing the symptoms and limitations at work<br>A4c. State that managing symptoms and limitations will help in staying productive and prevent progression of symptoms	S4a. Express confidence in the ability to recognize symptoms and limitations<br>S4b. Express confidence in the ability to manage the symptoms and limitations at work<br>S4c. List the symptoms that require actions<br>S4d. Recognizes problems in managing symptoms and limitations at work	P4a. Recognize that the employer is positive about managing symptoms and limitations at work<br>P4b. Feel support from employer and colleagues to manage symptoms and limitations at work

Table 2 An example of a matrix of change objectives for OPs

**Environmental outcome: occupational physicians collaborate with the work environment in supporting employees with chronic conditions to exert self-control**			
**Performance objectives**	**Personal determinants**		
	**Attitude**	**Skills & Self-efficacy**	**Perceived norm**
1. OP creates awareness within the organization of the need for an organizational policy to facilitate employees with chronic conditions staying at work	A1a. Express positive feelings about creating awareness within the organization for the need of an organizational policy	S1a. Express confidence about identifying relevant stakeholders in the organization<br>S1b. Express confidence in getting across the importance of an organizational policy to	P1a. Recognize that the employer is positive about exerting self-control by employees with chronic conditions

 A.R. Bosma[1], C.R.L. Boot[1], F.G. Schaafsma[1], G. Kok[2], J.R. Anema[1]; [1] Department of Public and Occupational Health, Amsterdam Public Health Research Institute, Amsterdam UMC, VU University Amsterdam, Amsterdam, The Netherlands. [2] Faculty of Psychology and Neuroscience, Maastricht University, Maastricht, the Netherlands; a.bosma@amsterdamumc.nl

		relevant stakeholders in the organization	
2. OP guides the employer in exploring organizational barriers which inhibit employees with chronic conditions from exerting self-control	A2a. Express positive feelings about identifying barriers for exerting self-control by employees with chronic conditions<br>A2b. State that identifying barriers for exerting self-control by employees with chronic conditions is the first step in creating an organizational policy	S2a. Express confidence about the ability to guide the employer with identifying barriers for exerting self-control by employees with chronic conditions	P2a. Recognize that the employer is positive about identifying barriers for exerting self-control by employees with chronic conditions<br>P2b. Feel support from employer in identifying barriers for exerting self-control by employees with chronic conditions
3. OP guides the employer in exploring possible solutions for these organizational barriers which inhibit employees with chronic conditions from exerting self-control	A3a. Express positive feelings about finding effective solutions for identified barriers<br>A3b. State that these solutions can help with exerting self-control by employees with chronic conditions and can help in creating an organizational policy	S3a. Express confidence about the ability to guide the employer with thinking up effective solutions	P3a. Recognize that the employer is positive about thinking up solutions for exerting self-control by employees with chronic conditions<br>P3b. Feel support from employer in thinking up solutions for exerting self-control by employees with chronic conditions
4. OP helps to create an organizational policy and a supportive work environment to facilitate the ability of employees with chronic conditions to exert self-control and stay at work	A4a. Express positive feelings about helping employer in creating an organizational policy<br>A4b. State that an organizational policy can facilitate exerting self-control by employees with chronic conditions	S4a. Express confidence in the ability to help create an organizational policy together with employer	P4a. Recognize that the employer is positive about creating an organizational policy

 A.R. Bosma[1], C.R.L. Boot[1], F.G. Schaafsma[1], G. Kok[2], J.R. Anema[1]; [1] Department of Public and Occupational Health, Amsterdam Public Health Research Institute, Amsterdam UMC, VU University Amsterdam, Amsterdam, The Netherlands. [2] Faculty of Psychology and Neuroscience, Maastricht University, Maastricht, the Netherlands; a.bosma@amsterdamumc.nl
