# Supplementary material for: Development of an Intervention to Create a Supportive Work Environment for Employees with Chronic Conditions: An Intervention Mapping Approach
Source: J Occup Rehabil. 2020 Mar 21;30(4):624–34. doi: 10.1007/s10926-020-09885-z (PMC7716852; doi:10.1007/s10926-020-09885-z)
Supplement: Supplementary file 3 — Supplementary file3 (PDF 182 kb) [file 10926_2020_9885_MOESM3_ESM.pdf]

Online resource 3

Table 1 Theoretical methods for the selected determinants

**Change objective (per determinant)**	**Theoretical methods**	**Parameters**	**Practical applications**
CO A1-4	Shifting perspectives	Requires social and organizational skills	OPs form workgroups and organize meetings with different stakeholders (e.g. employees, HR managers and supervisors) in an organization
CO A1-4	Environmental re-evaluation	Stimulation of cognitive as well as affective appraisal for improving appraisal and empathy skills	Stakeholders in the workgroup discuss the benefits of exerting self-control at work for employees with chronic conditions and their employers
CO A2; S2; P2	Organizational diagnosis and feedback	Requires consciousness raising, feedback and communication skills	The workgroup discusses and identifies potential barriers to exert self-control within their organization from different stakeholders' perspectives
CO A3; S3; P3	Participatory problem solving	Requires willingness to accept working group participants as equals; requires creative thinking and appropriate motivation	The workgroup discusses and brainstorms about possible solutions for these barriers to better support their employees in exerting self-control within their organization
CO A4; S4; P4	Structural redesign<br><br>Systems change	Requires management authority and agreement<br><br>Methods and actors depend on the level of the system	Agreed solutions are implemented in new or existing policy within a certain timeframe

Development of an intervention to create a supportive work environment for employees with chronic conditions: an Intervention Mapping approach; Journal of Occupational Rehabilitation; A.R. Bosma[1], C.R.L. Boot[1], F.G. Schaafsma[1], G. Kok[2], J.R. Anema[1]; [1] Department of Public and Occupational Health, Amsterdam Public Health Research Institute, Amsterdam UMC, VU University Amsterdam, Amsterdam, The Netherlands. [2] Faculty of Psychology and Neuroscience, Maastricht University, Maastricht, the Netherlands; a.bosma@amsterdamumc.nl
