# Supplementary material for: Development of an Intervention to Create a Supportive Work Environment for Employees with Chronic Conditions: An Intervention Mapping Approach
Source: J Occup Rehabil. 2020 Mar 21;30(4):624–34. doi: 10.1007/s10926-020-09885-z (PMC7716852; doi:10.1007/s10926-020-09885-z)
Supplement: Supplementary file 4 — Supplementary file4 (PDF 349 kb) [file 10926_2020_9885_MOESM4_ESM.pdf]

Online resource 4

Fig 1. Examples of forms, used to guide the Participatory Approach

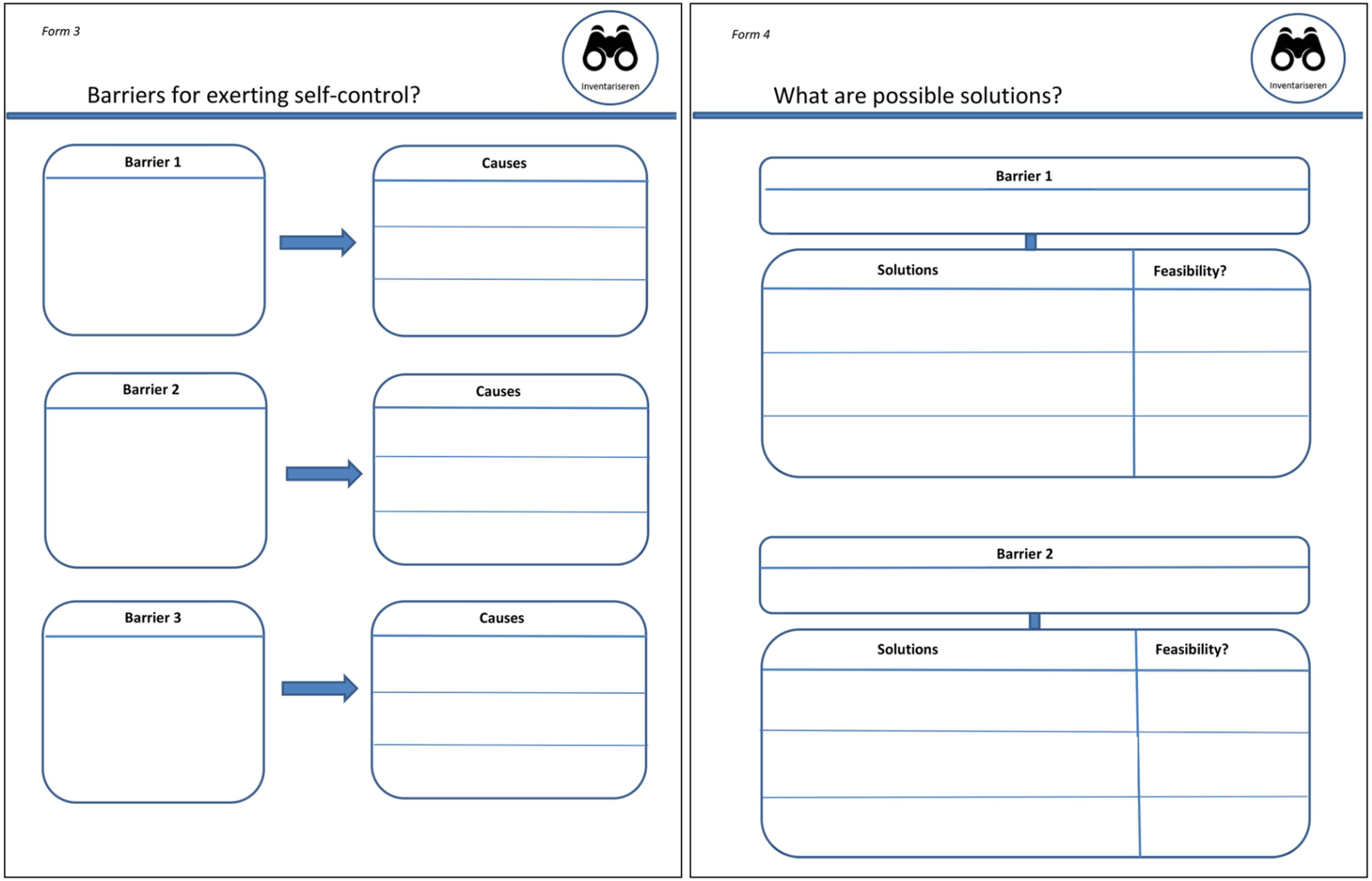

Development of an intervention to create a supportive work environment for employees with chronic conditions: an Intervention Mapping approach; Journal of Occupational Rehabilitation; A.R. Bosma[1], C.R.L. Boot[1], F.G. Schaafsma[1], G. Kok[2], J.R. Anema[1]; [1] Department of Public and Occupational Health, Amsterdam Public Health Research Institute, Amsterdam UMC, VU University Amsterdam, Amsterdam, The Netherlands. [2] Faculty of Psychology and Neuroscience, Maastricht University, Maastricht, the Netherlands; a.bosma@amsterdamumc.nl
